# A neural pathomics framework for classifying colorectal cancer histopathology images based on wavelet multi-scale texture analysis

**DOI:** 10.1038/s41598-021-94781-6

**Published:** 2021-07-30

**Authors:** Eleftherios Trivizakis, Georgios S. Ioannidis, Ioannis Souglakos, Apostolos H. Karantanas, Maria Tzardi, Kostas Marias

**Affiliations:** 1 Medical School, University of Crete, 71003 Heraklion, Greece; 2 Computational Biomedicine Laboratory (CBML), Foundation for Research and Technology Hellas (FORTH), 70013 Heraklion, Greece; 3 Laboratory of Translational Oncology, Medical School, University of Crete, 71003 Heraklion, Greece; 4 Department of Medical Oncology, University Hospital of Heraklion, 71500 Heraklion, Greece; 5 Department of Radiology, Medical School, University of Crete, 71003 Heraklion, Greece; 6 Electrical & Computer Engineering, Hellenic Mediterranean University, 71410 Heraklion, Greece

**Keywords:** Diagnostic markers, Information technology, Computer science

## Abstract

Colorectal cancer (CRC) constitutes the third most commonly diagnosed cancer in males and the second in females. Precise histopathological classification of CRC tissue pathology is the cornerstone not only for diagnosis but also for patients’ management decision making. An automated system able to accurately classify different CRC tissue regions may increase diagnostic precision and alleviate clinical workload. However, tissue classification is a challenging task due to the variability in morphological and textural characteristics present in histopathology images. In this study, an artificial neural network was trained to classify between eight classes of CRC tissue image patches derived from a public dataset with 5000 CRC histopathology image tiles. A total of 532 multi-level pathomics features examined at different scales were extracted by visual descriptors such as local binary patterns, wavelet transforms and Gabor filters. An exhaustive evaluation involving a variety of wavelet families and parameters was performed in order to shed light on the impact of scale on pathomics based CRC tissue differentiation. Our model achieved a performance accuracy of 95.3% with tenfold cross validation demonstrating superior performance compared to 87.4% reported in recent studies. Furthermore, we experimentally showed that the first and the second levels of the wavelet approximations can be used without compromising classification performance.

## Introduction

Pathomics are features stemming from the analysis of digitized histopathology images with the use of image analysis methods that extract sub-visual attributes for characterizing disease appearance and behavior. As in radiomics, the main image processing methodology used to extract a large number of characteristic image features is texture analysis. However, is it well known that texture may exist in different scales^1^ and for this reason wavelet transforms have been used to characterize tissue properties as part of artificial intelligence and radiomics pipelines. The main rationale for using wavelets is their ability to decipher textural information from different scales, which has been shown to significantly improve image classification^2^. However, the impact regarding the choice of wavelet families or level of decomposition on the performance of radiomics/pathomics methods, remains a poorly addressed topic in the literature. Wavelets are widely used in many fields of research such as time-series analysis, signal denoising^3^ and filtering for radiomics analysis in medical imaging^4^. In particular, Sharma et al.^5^ investigated a feature extraction method based on different multi-resolution schemes by incorporating wavelet packet transform (WPT) and Gabor wavelet transform (GWT) over the ROIs (Regions of Interest) of breast ultrasound images. The examined dataset comprised 167 cases of breast lesions including 60 fibro-adenoma, 50 carcinomas and 57 metastases. A support vector machine (SVM) classifier was used for differentiating among breast lesions yielding accuracy (ACC) up-to 89.5% and sensitivity (SN) up-to 95.5%. Wang et al.^6^ studied 3 datasets with a total of 381 T2-weighted brain images for pathological brain detection (PBD). Discrete Wavelet Packet Transform (DWPT) of the Haar wavelet family and Tsallis entropy (TE) was used for feature extraction. In particular, sixteen wavelet sub-bands were extracted from the original image and TE was calculated on sub-band coefficients. The classification of breast lesions was performed by a Feed-Forward Neural Network (FNN) trained on DWPT-TE features and achieving a performance accuracy of 99.5%. In another study^7^, the WPT of the Daubechies family was examined as a pre-processing method for the B-mode ultrasound liver images over the RF frames. Statistical and texture features such as first-order gray level parameters, gray-level co-occurrence matrix (GLCM), and local binary patterns (LBP) were calculated and machine-learning selection and classification was applied. The author claims a significant reduction in computational time up-to 75% with a cost of only 1% in terms of performance accuracy. Takruri et al.^8^ proposed a feature extraction methodology by combining the energy of multiple wavelet coefficients from melanoma histopathology images, achieving a performance accuracy of 87.1%.

Some noteworthy studies on the examined histopathology dataset^9^, introduced machine and deep learning analyses on this challenging multi-class problem. A variety of imaging features (GLCM, LBP, Gabor, histogram) and machine learning classifiers (SVM, decision trees, nearest neighbor) were examined, achieving an accuracy of 87.4%^10^. Cascianelli et al.^11^ investigated a pre-trained “off-the-shelf” deep learning (ACC 84%) and machine learning approaches (ACC 72.8%-79.6%) with texture spectrum features and principal component analysis (PCA) over a wide range of datasets including the examined. Sarkar et al.^12^ proposed a saliency guided dictionary learning (SDL) framework achieving ACC from 51.1 to 73.7% for a 7-class subset of the original set excluding patches from the background class. However, it should be noted that these studies did not follow a multi-scale approach in feature extraction which can be the reason for the limited performance reported.

In this paper, the WPT was used both as a composite pre-processing (filtering), image decomposition scheme and as a feature extraction methodology for improving the performance and accelerating the convergence of machine learning and artificial neural network (ANN) models. It is noteworthy that in oncology imaging studies with wavelets^13–18^ the impact of different families or level of decomposition on the performance of their experimental methods is rarely investigated. The lack of such an analysis highlights a critical scientific question regarding the optimal use of wavelets in the context of radiomics or pathomics approaches in medical image analysis in order to evaluate the effectiveness of image features at different frequency bands. To this end, an exhaustive multi-scale pathomics analysis integrating WPT filtering with all available wavelet transforms and different levels of decomposition was conducted to identify the optimal set of level, sub-band and family for improved CRC tissue differentiation. In particular, improvements such as lower computational cost of the analysis and increased classification performance (up-to ACC 95.3%) were observed in the examined histopathology dataset compared to the current state-of-the-art literature^10^ (ACC 87.4%). The main contribution of the presented paper is the first in-depth application of wavelet analysis for pathomics-based multi-class pathology tissue classification, investigating the role of scale through wavelet image decomposition and achieving improved performance compared to the state-of-the-art methods.

## Results

**Figure 1:**
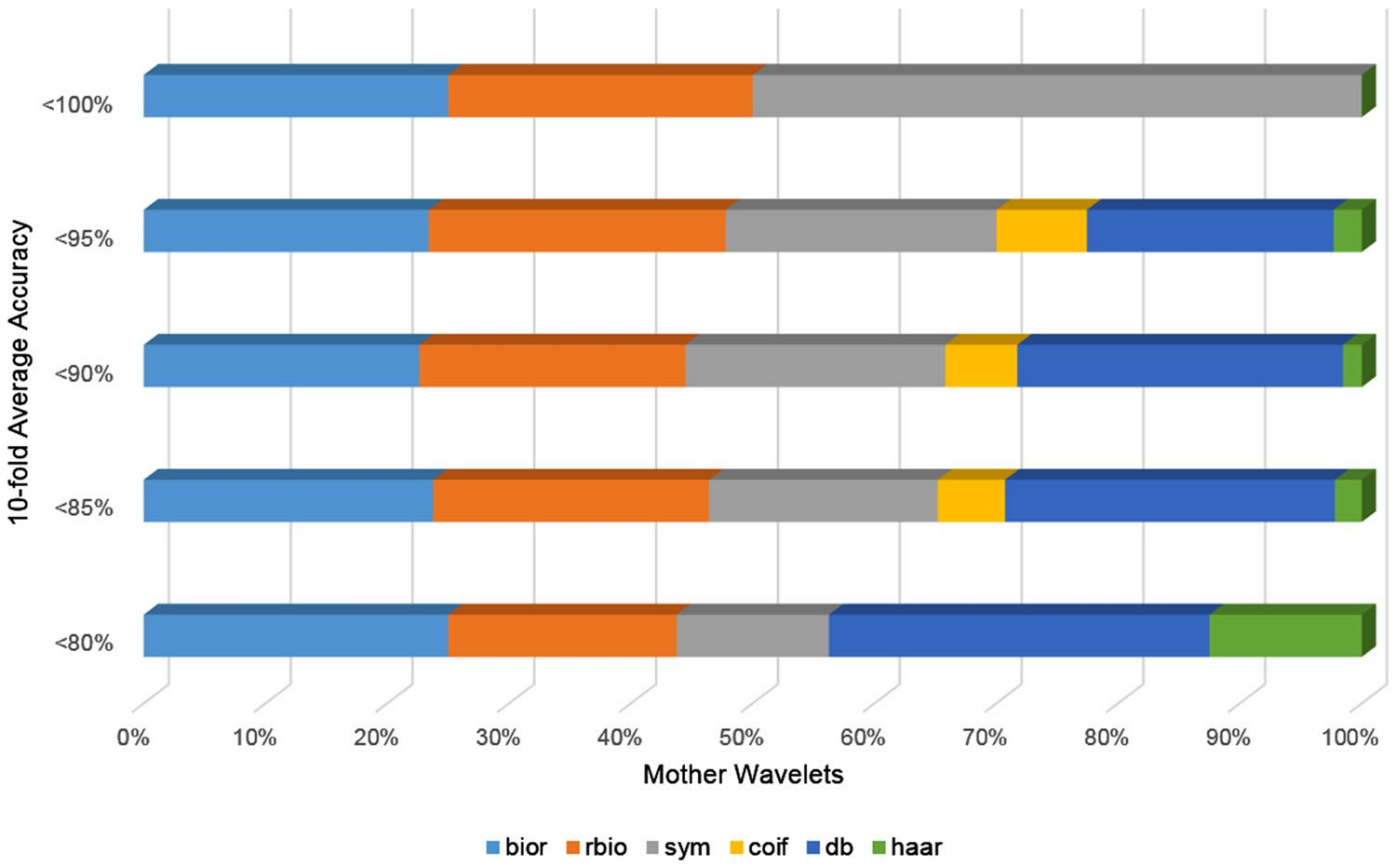
Performance analysis of wavelet families: Each line shows the percentage of each mother wavelet with respect to the total number of models achieving different levels of accuracy. *db* daubechies, *sym* symlets, *coif* coiflets, *bior* biorthogonal, *rbio* reverse biorthogonal. (Figure created in Excel 2013, https://www.microsoft.com/microsoft-365/excel).

**Figure 2:**
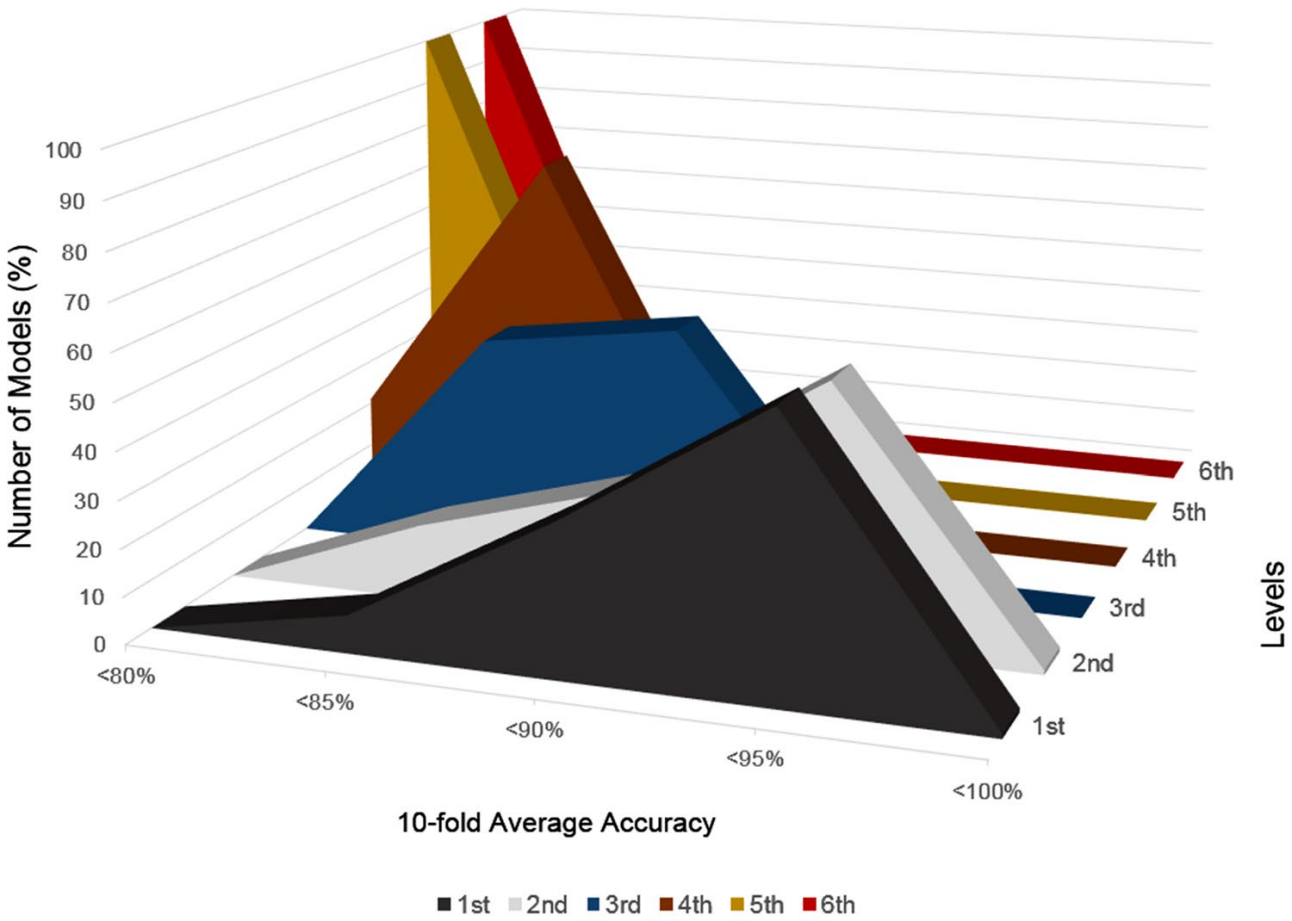
Performance analysis among different levels of wavelet packet transform: the first and second levels exhibit high accuracies while in higher levels of decomposition the texture analysis error is significantly affected. (Figure created in Excel 2013, https://www.microsoft.com/microsoft-365/excel).

Sample selection bias is a common risk when applying machine learning techniques, particularly in ANN. This can lead to a compromised model with limited generalization capacity. A hold-out method was performed on the full dataset for randomly selecting the 10% of the images as a validation set for applying feature selection on the SVM experiments and hyperparameter optimization during the ANN convergence. In addition to the hold-out method, an iterative tenfold cross-validation process was applied on the rest 90% for splitting the images into a training set for model fitting and an unseen testing set per split for model evaluation. In particular, the validation set consisted of 500 images and at each cross-validation split, 4050 images were used as a training set and 450 for the testing set. The class balance across all sets was preserved to assess an unbiased performance analysis. There are 625 tissue samples (12.5%) of each of the eight categories in the examined colorectal dataset. Maintaining these ratios in the smaller validation and testing sets is important for both the feature selection and model evaluation tasks respectively, because it ensures that each class is represented equally and no bias is introduced from the data stratification process. The studied tissue tiles were subjected to wavelet decomposition up to six layers and using the six discrete wavelet families (bior, rbio, db, sym, haar, and coif), generating 378 sets of approximated images. A corresponding number of sets of imaging features were calculated from the WPT images. This resulted in the same number of models per analysis pipeline (baseline SVM, selected features with SVM and ANN). A multi-level feature extraction method (Fig. 4) was applied on the decomposed wavelet images. A total of 532 features comprised of first order statistics of pixel intensities, local binary patterns, and gabor filtered images combined with gray level co-occurrence matrices, and higher order features such as contrast, dissimilarity, homogeneity, correlation, angular second moment, and energy. A subset of these features were selected based on the two criteria: (a) the statistical significance of F with a p-value less than 0.05 and (b) the F should be greater than the F_critical value_. The F_critical value_ was calculated by the percent point function^19^ using the aforementioned threshold in conjunction with the degrees of freedom of the dataset. It should be noted that the best features were comprised of first order statistics over the raw wavelet and gabor image (4 pixels per circle), local binary pattern features with finer quantization of the angular space, energy, dissimilarity, correlation, angular second moment from grey level features with a distance of one pixel. Three classification strategies were applied: support vector machines with radial basis function trained on full pathomics or selected pathomics and artificial neural networks trained on full pathomics. Only two parameters were variable across each analysis pipeline: the mother wavelet and the level of decomposition. Hyper-parameter optimization (number of layers, number of neurons, activation functions) was applied to assess the fitting status and mitigate the overfitting of the ANN. On average, more than 200 epochs were required for the ANN model to converge. The use of an early-stopping technique, in which the training is terminated when the validation error reaches a global minima, aided in preventing model overtraining. A few thousand models were evaluated during the experimental phase of this study. The computational infrastructure used during the experiments comprises an 8-core i7 processor with 24 gigabytes of RAM and a GTX 1070 graphics processor with 8 gigabytes of VRAM. Approximately 150 hours required for the proposed feature extraction process to complete on the entire dataset of 5000 colorectal image patches. This includes the calculation of all the examined wavelet families with up-to six levels of decomposition, selection and the extraction of the multi-level features. Additionally, 20 hours required for the SVM classification (with and without feature selection) and approximately 40 hours for the ANN convergence and evaluation. The SVM-based models (with and without feature selection) achieved accuracy up-to 84%, which is comparable to the published “off-the-shelf” deep learning approach^11^. It is worth noting that models based on ANNs scored the highest performance (up-to ACC 95.3%), particularly in the second level of approximation of symlet wavelet family, as evident by Table 1. A comprehensive analysis of the performance grouped by wavelet family is presented in Fig. 1 and by the level of decomposition in Fig. 2. Additional resources such as the source code and the corresponding optimized parameters can be made available upon request.

**Table 1:** Performance analysis of the current literature in comparison to the tenfold average accuracy of the examined SVM-based and ANN models. Bold font indicates the best performance.

	State-of-the-art		Method	Input features	ACC (%)
Literature	Kather et al.^10^		SVM	GLCM, LBP, Gabor	87.4
	Cascianelli et al.^11^		NNC/VGG-based “off-the-shelf”	LBP, Texture Spectrum, PCA / Deep Features	79.60/84.00
	Sarkar et al.^12^		SDL	Local Gabor filtering	73.7

	WPT family		Levels	# Features	ACC (avg ± std%)
Baseline SVM	rbio		1	155	84.04 ± 1.98
	bior		2	155	83.22 ± 2.34
	coif		2	140	83.21 ± 1.24
	sym		1	135	83.04 ± 1.63
	rbio		2	532	82.76 ± 1.67
	bior		2	532	82.18 ± 1.35
	coif		2	532	82.16 ± 1.57
	db		2	532	82.10 ± 1.68

	Artificial neural network		Input layers	Hidden layers	ACC (avg ± std%)
Neural pathomics	Number of neurons	64	532	1 × sigmoid	87.84 ± 1.16
		128			90.66 ± 1.23
		256			92.46 ± 1.24
		512			94.34 ± 0.77
		1024			94.91 ± 0.91
		2048			93.54 ± 2.31
		64	532	2 × sigmoid	86.4 ± 1.58
		128			89.68 ± 1.84
		256			92.77 ± 1.71
		512			94.56 ± 2.01
		1024			**95.32 ± 2.16**
		2048			94.13 ± 1.94

## Discussion

To date, wavelet decomposition has been used for feature extraction or as a filtering method in medical image analysis and radiomics but the selection of its parameters has not been thoroughly investigated^13–18^ which is indicative of the lack of research on the role of scale in most radiomics and pathomics studies. Since wavelet image transforms produce a series of band-pass outputs at each scale, i.e. the wavelet coefficients, there is a need for an exhaustive analysis for determining the impact of scale representation in the feature extraction process and the performance of the developed models. Depending on the application, different frequency bands might provide more invariant image information and therefore salient features for classification. In this study, we presented a pathomics methodology for addressing a challenging multiclass CRC tissue classification problem on an open-access dataset while investigating in parallel the impact of different mother wavelets on the discriminative power of the proposed analysis in order to incorporate optimal, scale-related information within the proposed pathomics pipeline. The WPT approximation pipeline enabled a low classification error despite projecting the original image to a much lower spatial resolution. Biorthogonal, reverse biorthogonal and symlets were among the best performing mother wavelets across all ANN models for this pathomics classification problem. Notably, the same three families were among the most accurate SVM models according to Table 1. Overall, ANN models achieved the highest accuracy because of their superior fully-automated selection and combination of pathomic features. The state-of-the-art machine learning analysis^10^ on full image tile resolution (150 by 150 pixels) performed significantly lower than the proposed methodology (ACC 87.4% versus 95.3%), as shown in Table 1. The reduced spatial dimensionality is a significant advantage of this approach (at least for the first and second level) with the examined samples spanning across 39 by 39 to 79 by 79 pixels in contrast to an analysis with full pixel array resolution. Despite this substantial reduction in spatial information prior to feature extraction, the proposed SVM analysis achieves similar performance to the state-of-the-art approaches (Table 1, baseline SVM 84% versus 87.4^10^/79.6-84^11^/73.7^12^%). ANN models were introduced to address the limitations of a feature-based selection process that was followed by the proposed SVM and examined studies^10–12^, enabling a fully automated analysis of the complete pathomics feature vector. As a result, a significant reduction in terms of classification error was achieved. The use of WPT in the proposed pathomics classification approach led to an efficient feature extraction pipeline exploiting a variety of mother wavelet transformations with lower computational complexity. Nevertheless, the prediction error increased when deeper levels of WPT decomposition were applied regardless of the mother wavelet, feature extraction or classification method. This effect can be observed in Fig. 2 and can be attributed to the reduced spatial resolution and information content of the approximated image which depends on the applied level of decomposition. The execution times of the WPT-based over the original image tiles were improved on average from 28.4% up to 49% per image depending on the level of decomposition, as reported in

Table 2 Additionally, it is worth noting that in this study the full dataset with all 8 classes was used for the proposed analysis while Sarkar et al.^12^ excluded the background class. The first and the second decomposition levels were found (Fig. 2) to perform best compared to the higher levels of scale. This can be explained by the significant loss of spatial information after the second level of WPT, even below the previously mentioned 39 by 39 pixels. The insights from the parameter optimization process of WPT (Figs. 1, 2) can be useful for image analysis tasks that deal with complex tissue differentiation as for example in radiomic applications with wavelet filtering. Additionally, the encouraging results of the presented study could be the first step towards an automated annotation system providing a fast method for patch extraction over multiple high resolution histopathology images with no manual pixel- or patch-based annotations available. A few potential limitations of WPT for image analysis include the non-intuitive parameter optimization process involving mother wavelets, sub-bands and level of decomposition. Additionally, the feature selection process and the lack of a well-founded process for optimal combination of the best sub-bands or high-level features can render such analysis hard to perform. An interesting endeavor in future studies would be to analyze the impact of wavelet filtering and decomposition on deep learning convergence or as a data augmentation technique with the additional benefits of enhanced feature engineering. Sub-class discrimination for oncology and radio-, patho-genomics could also be attractive application domains for further research on the use of wavelets transformations as a mean to augment information extraction in a multi-scale fashion.

**Table 2:** Execution times of the proposed feature extraction methodology for the original image and different levels of WPT.

Wavelet—level	Pixel array size (pixels)	Time per image (avg ± std second)
Original tile	150 by 150	1.41 ± 0.26
Bior1.1—1	75 by 75	1.01 ± 0.16
Bior1.1—2	38 by 38	0.81 ± 0.22
Bior1.1—3	19 by 19	0.75 ± 0.11
Bior1.1—4	10 by 10	0.72 ± 0.09
Bior1.1—5	5 by 5	0.72 ± 0.08
Bior1.1—6	3 by 3	0.71 ± 0.12

In this study, an extensive optimization of WPT parameters was implemented and significant improvements in terms of discrimination accuracy for the examined CRC tissue classification problem were achieved. This is in line with our initial working hypothesis that texture is a scale-dependent phenomenon that may significantly affect performance in radiomics/pathonics applications. Our results indicate that proper customization of a wavelet analysis should be prioritized in the design of a pathomics computational pipeline. The mother wavelet and sub-band selection can affect the classification performance of the analysis, thus providing a multi-resolution framework for implicit feature engineering. Furthermore, efficiency in computational cost of the extraction of complex features can be achieved with the projection into a lower dimensional space through WPT decomposition. Our results also confirm that ANNs with their superior fully-automated analysis can address the shortcomings of other machine learning techniques in terms of feature selection, combination and differentiation. The results of the current study should be tested in independent cohorts and in correlation with genomics findings and patients’ outcome.

## Methods

### Histopathology images dataset

**Figure 3:**
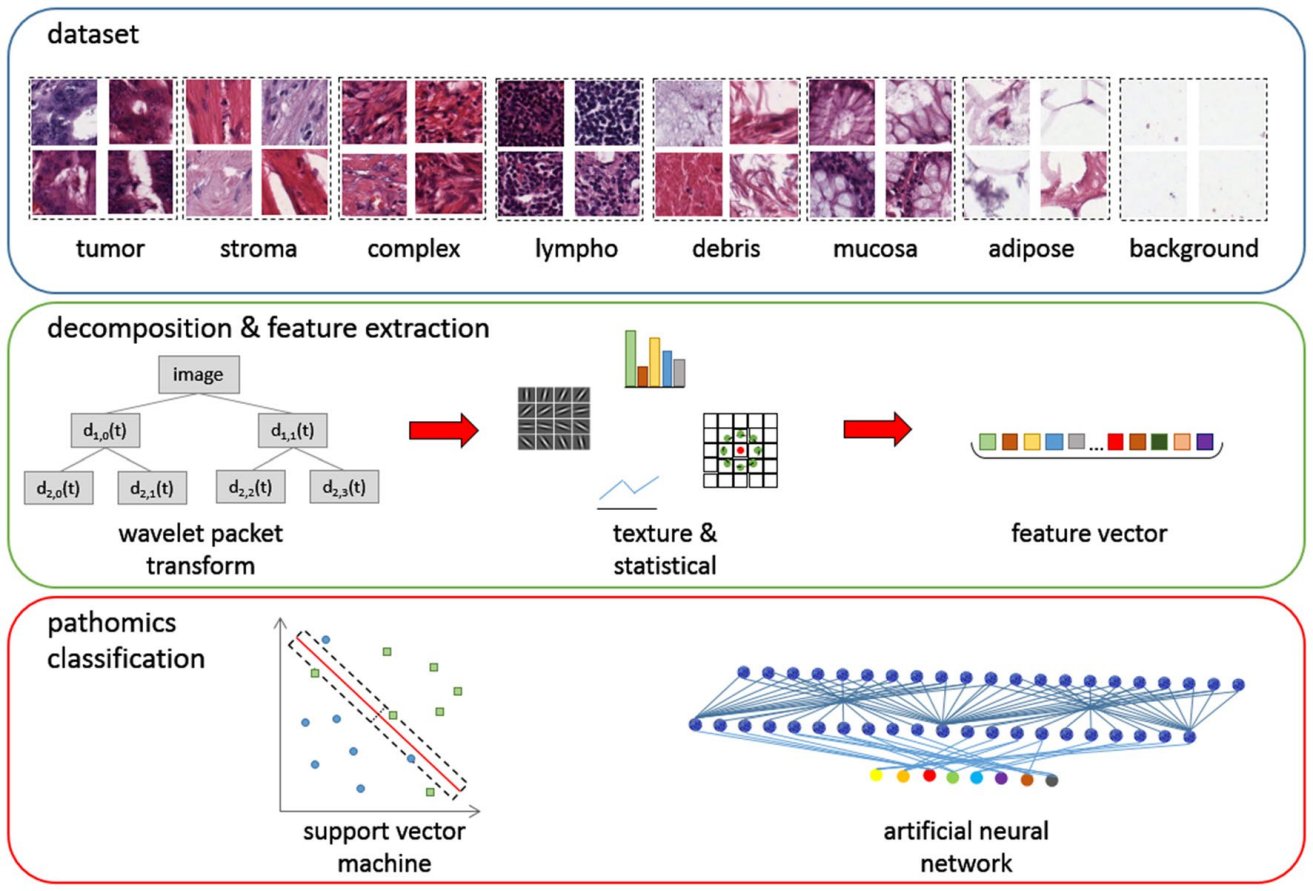
Samples from the examined dataset and the proposed multi-level textural analysis pipeline featuring wavelet packet transform, local binary patterns, Gabor, grey level co-occurrence matrices, first order statistics concatenated in a pathomics signature for support vector machines and artificial neural networks tissue classification. (Figure created in PowerPoint 2013, https://www.microsoft.com/microsoft-365/powerpoint).

The examined colorectal cancer dataset^9^ titled “Collection of textures in colorectal cancer histology” is available online as an open-access repository via the following link (https://doi.org/10.5281/zenodo.53169). The formalin-fixed paraffin-embedded colorectal primary tumor tiles were gathered from the University Medical Center Mannheim (Heidelberg University) in Germany. Manual extraction of 5000 patches with 74 by 74 μm dimension (0.495 μm per pixel) was performed in a set of ten digitized hematoxylin & eosin (H&E) stained tissue slides. In particular, the patches were organized into eight classes (Fig. 3) including seven tissue types from the tumor epithelium, simple stroma, complex stroma, lymphoid follicles, debris, mucosal glands, adipose and background patches with no tissue.

### Experimental protocol

**Figure 4:**
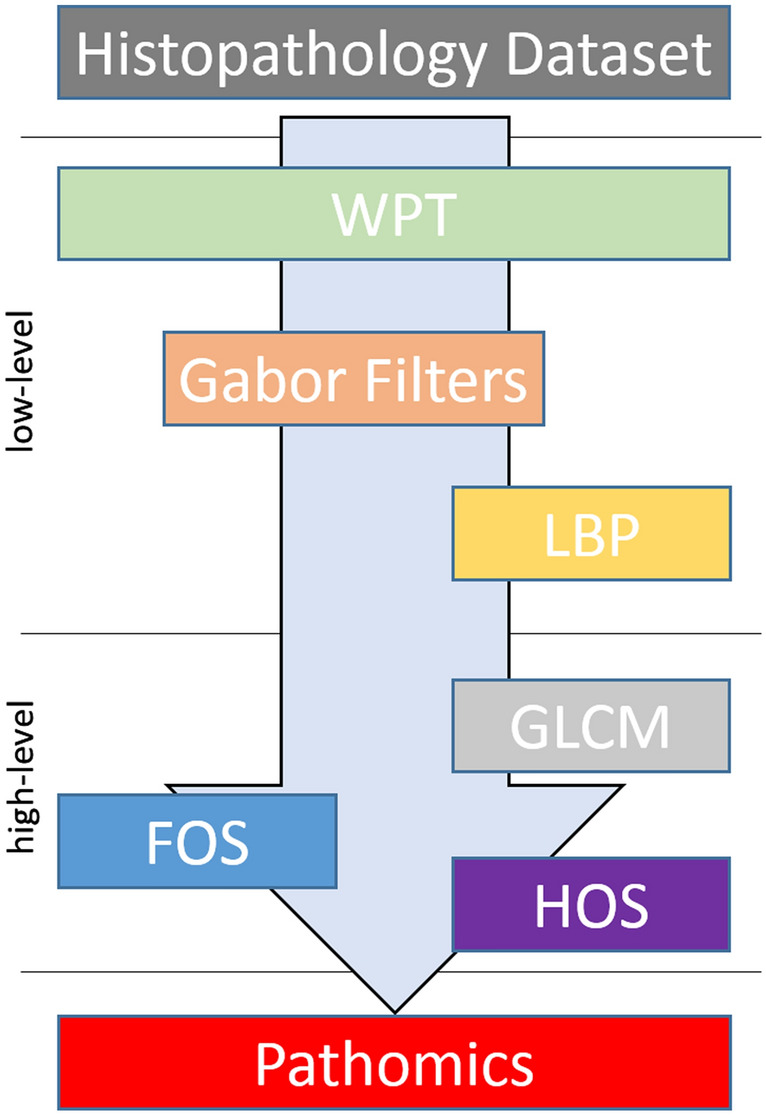
The proposed multi-level feature extraction strategy including several combinations of WPT, Gabor filters, LBP, GLCM, first and high order statistics. *WPT* wavelet packet transform, *LBP* local binary patterns, *GLCM* grey level co-occurrence matrices, *FOS* first order statistics, *HOS* higher order statistics. (Figure created in PowerPoint 2013, https://www.microsoft.com/microsoft-365/powerpoint).

The wide variety of cell and tissue types in the studied dataset constitute a complex classification problem and was the main motivation for proposing a framework for investigating the impact of multi-scale wavelet transformations in terms of texture analysis. WPT was applied on the histopathology tiles using every discrete mother wavelet and level of decomposition available in the pywavelets library^20^. In particular, six families of wavelets with a total of 105 variations and up-to 6 levels of decompositions were applied on the original tissue tiles and resulting in 378 datasets of approximated images were calculated. For each one of these derived datasets with different wavelet setting, a composite feature extraction process was executed as is illustrated in Fig. 4. The features included multiple combinations of texture-based features (WPT-LBP, WPT-Gabor filters, WPT-GLCM, WPT-LBP-GLCM, WPT-Gabor-GLCM, and other as depicted in Fig. 4), first order statistics (FOS) and higher order statistics (HOS) as described in the “Statistical features” section below. Thus, the resulted pathomics vector consisted of 532 features extracted from each level of decomposition in the proposed analysis (Fig. 4). The overall analysis is presented in Fig. 3. “Best practices” in machine learning analysis^21^ were considered mainly by applying a class-preserving hold-out method with 10% of the full dataset for determining a subset of pathomics for feature selection (SVM analysis) and hyperparameter optimization/early stopping (ANN analysis). Additionally, on the remaining images a 10-fold cross-validation was performed iteratively for splitting into training and unseen testing sets. The three separate sets ensured a fair and unbiased model evaluation. In total, three different classification strategies were performed, two based on SVMs and one on ANN. The SVMs were applied on the full pathomics vector and on a set of statistical significant features. An extensive hyperparameter optimization was performed to identify the best configuration of ANN, including the learned parameters (number of neurons and layers) and other parameters such as learning rate, dropout rate, activation functions and optimizer. The ANN models were trained on the 532-pathomics vector.

### Decomposition and feature extraction

The proposed feature extraction approach is comprised of individual operators such as WPT, Gabor, LBP and GLCM paired with first-order statistics and combinations of higher order statistics methods. This diverse set of simple and complex features with multiple levels of analysis aims in forming a discriminative and robust pathomics signature. The final vector consists of 532 imaging features. A detailed depiction of the proposed multi-level feature extraction methodology is provided in Fig. 4.

#### Wavelet packet transform (WPT)

Since a thorough description of the mathematical background is demanding and beyond the scope of this work, we briefly, present the one dimensional continuous wavelet transform below. Further in depth information regarding the two dimensional discrete wavelet transform applied to images can be found in literarure^22,23^.

Assume a function f(t) in the set of square integrable functions ${L}^{2}({\mathbb{R}})$. A continuous wavelet transform (CWT) shows how f(t) is decomposed into a set of basis functions g(t), called wavelets, and is given by:

1 $$CW{T}_{g}f\left(s, \tau \right)= \underset{-\infty }{\overset{\infty }{\int }}f\left(t\right){g}_{s,t}^{*}\left(t\right) dt$$

In Eq. (1), *represents the complex conjugate; $s,\tau$ are the variables related to scale and translation after the transform and are typically the new dimensions. In the generalized case of two dimensional CWT a scaling function and three two dimensional wavelets are compulsory which are responsible for functional variations (image intensity changes) along different directions such as variations along columns (horizontal edges), variations along rows (vertical edges) and variations along diagonals giving the sense of image texture.

**Figure 5:**
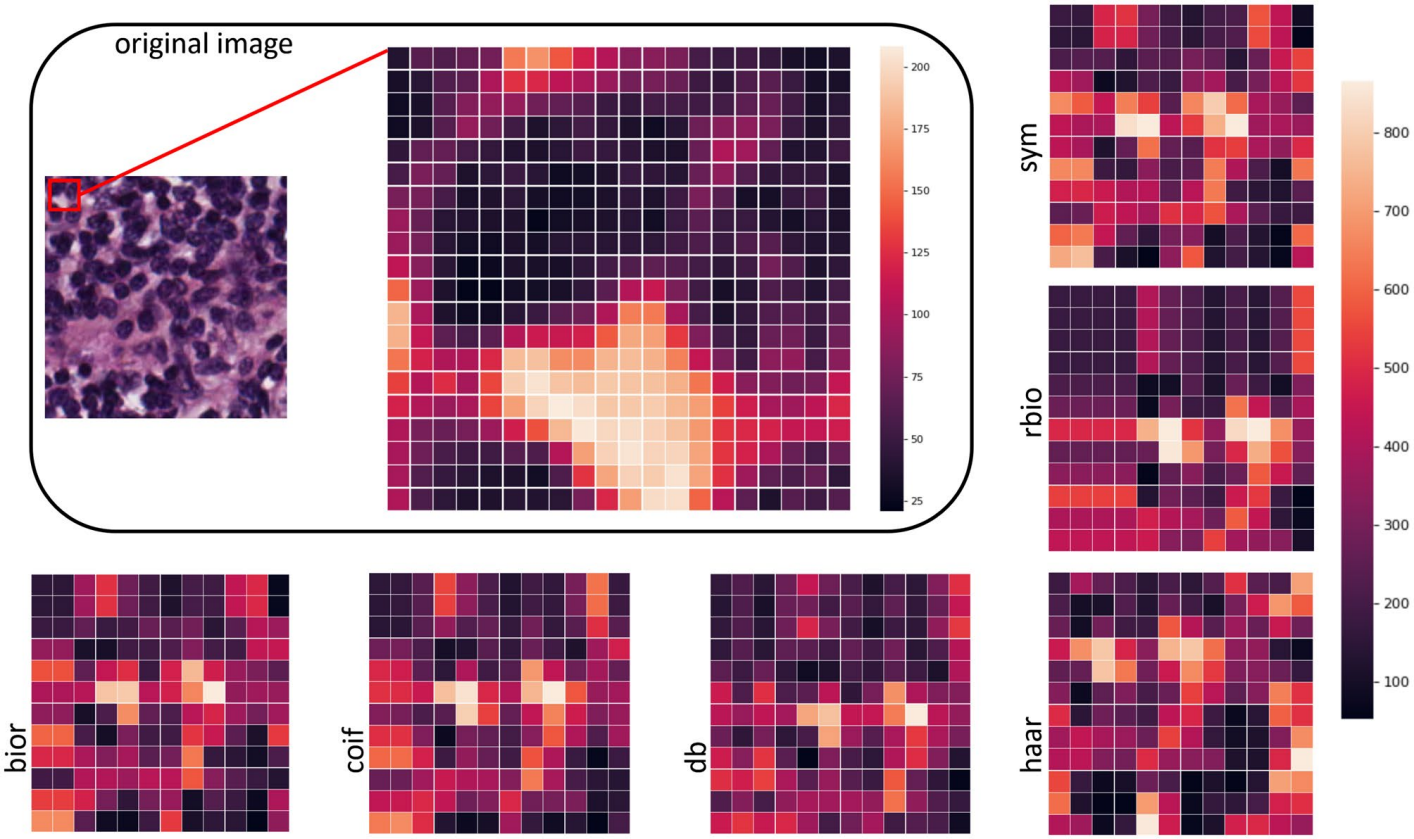
Differences among mother wavelets at the second level of decomposition for part (red bounding box) of the original image. *db* daubechies, *sym* symlets, *coif* coiflets, *bior* biorthogonal, *rbio* reverse biorthogonal. (Figure created in PowerPoint 2013, https://www.microsoft.com/microsoft-365/powerpoint).

To explore textural information from the studied histopathology images a variety of bases of discrete mother wavelets were used from the pywavelets library such as Haar (haar), Daubechies (db), Symlets (sym), Coiflets (coif), Biorthogonal (bior), and Reverse biorthogonal (rbio). Other families of such as Gaussian wavelets (gaus), Mexican hat wavelet (mexh), Morlet wavelet (morl), Complex Gaussian wavelets (cgau), Shannon wavelets (shan), Frequency B-Spline wavelets (fbsp), and Complex Morlet wavelets (cmor) wavelets were rejected because of their continuous nature. An indicative illustration of these wavelet transforms applied to a lymphoid follicles tile image is depicted in Fig. 5.

#### Gabor filters

The wavelet transformed image tiles were used as the input for filtering with multiple Gabor kernels^24^ calculated over five directions and four wavelengths from 4 to 20 pixels per cycle. Additionally, various rotation angles (from 70° to 190°) were employed on the original input image.

2 $${G}_{c\left[i,j\right]}=K{e}^{-\frac{({i}^{2}+{j}^{2})}{2{\sigma }^{2}}}\mathrm{cos}\left(2\pi f\left(icos\theta +jsin\theta \right)\right)$$

3 $${G}_{s[i,j]}=K{e}^{-\frac{({i}^{2}+{j}^{2})}{2{\sigma }^{2}}}sin(2\pi f\left(icos\theta +jsin\theta \right))$$

In Eqs. (2 and 3) K is the scale factor, f the frequency of the complex harmonic function, θ is the direction of the filter and σ the standard deviation in any filter direction.

#### Local binary patterns (LBP)

The LBP images were calculated over the WPT approximations with three methods including gray scale invariant, variance measures of the intensities of local texture, and uniform patterns with finer set points^25^. A radius of one pixel and with eight symmetric neighbors was selected for calculating this visual descriptor. The arrow path in Fig. 4 represents the integration of LBP images with statistical, Gabor and GLCM operators.

4 $${LBP}_{P,R}\left({x}_{c},{y}_{c}\right)=\sum_{P=0}^{P-1}s({i}_{P}-{i}_{c}){2}^{P}$$

5 $$s\left(x\right)=\left\{\begin{array}{c}1 if x\ge 0\\ 0 if x<0\end{array}\right.$$

In Eq. (4) P denotes the neighbor pixels, c the central pixel of the operator, i the gray-level value of their respective subscript, and s is defined in the fifth equation.

#### Statistical features

First order features were extracted over the approximated WPT, Gabor filtered or LBP images including metrics such as maximum, minimum, mean and standard deviation of pixel values of the feature maps. Gray Level Co-occurrence Matrix (GLCM) features are based on the co-occurrence of intensities of the pixels at a given direction and distance^26^. In particular, four angles from 0° to 135° and three distances from 1 to 5 pixels were calculated. A set of higher order statistical features such as contrast [Eq. (6)], dissimilarity [Eq. (7)], homogeneity [Eq. (8)], correlation [Eq. (9)], angular second moment (ASM) [Eq. (10)] and energy [Eq. (11)], were computed from the calculated co-occurrence matrices.

6 $$Contrast : {\sum }_{i,j=0}^{N-1}{P}_{i,j}{(i-j)}^{2}$$

7 $$Dissimilarity: {\sum }_{i,j=0}^{N-1}{P}_{i,j}|i-j|$$

8 $$Homogeneity: {\sum }_{i,j=0}^{N-1}\frac{{P}_{i,j}}{1+{(i-j)}^{2}}$$

9 $$Correlation: \sum_{i,j=0}^{N-1}{P}_{i,j}\left[\frac{(i-{\mu }_{i})(j-{\mu }_{j})}{\sqrt{({\sigma }_{i}^{2})({\sigma }_{j}^{2})}}\right]$$

10 $$ASM: {\sum }_{i,j=0}^{N-1}{P}_{i,j}^{2}$$

11 $$Energy: \sqrt{ASM}$$

In the above equations μ denotes mean, σ^2^ the variance of the intensities of the reference pixels, N is the number of gray levels and P_i,j_ the normalized GLCM element.

### Feature selection

The analysis of variance (ANOVA^27^ Test) was used for feature selection on the extracted pathomics in one SVM model of the presented analysis (the other used the full feature vector). The joint analysis of p-values with the corresponding F-values of the examined vector supported the identification of a subset of features establishing a compact and discriminative signature. This method was applied during the experimental phase and solely on the validation set reducing the dimensionality of feature vector. Based on these derived feature labels, the statistically significant components were then selected in both training and testing sets. The later remained unseen throughout this process. No feature selection method was applied during the ANN experiments.

### Classification

The classification was performed among eight classes including seven tissue types and the background of the tiles. Two different machine learning approaches for classification were applied: support vector machines and artificial neural networks. Multiple radial basis function (rbf) based SVM models were trained on selected and full pathomics to establish the baseline of the analysis. ANN were trained and evaluated on the complete pathomics signature as high-level data analysis models for end-to-end and fully-automated feature selection and classification. A cross-validation data stratification technique was applied to separate the dataset in ten parts one serving as the unseen testing set and the rest as the training set with iteratively interchangeable roles among the splits.

### Performance evaluation

The studied dataset is comprised of 8-classes, as discussed mentioned. Thus, the multiclass model was assessed by the classification accuracy where TP, TN, FP, and FN stand for true-positive, true-negative, false-positive and false-negative respectively. The performance metrics presented in the following sections refer to the average accuracy of the 10-fold cross-validation.

$$ACC= \frac{TP+TN}{TP+TN+FP+FN}$$
